# Deficiency of phosphatidylethanolamine synthesis: consequences for skeletal muscle

**DOI:** 10.1093/function/zqad044

**Published:** 2023-09-01

**Authors:** Robin Elaine Duncan

**Affiliations:** Department of Kinesiology and Health Sciences, University of Waterloo, Waterloo, ON N2L 3G1, Canada

**Keywords:** Pcyt2/ECT, phosphatidylethanolamine, skeletal muscle, myosteatosis, hepatosteatosis, insulin resistance, NAFLD

## A Perspective on “Skeletal Muscle Consequences of Phosphatidylethanolamine Synthesis Deficiency”

Phosphatidylethanolamine (PE) is a major glycerophospholipid (PL) in cellular and organellar membranes, and dysregulation of PE synthesis has been associated with energy over-storage and insulin resistance.^1^ Due to the small size of its ethanolamine headgroup relative to the volume occupied by its long and, in particular, unsaturated fatty acyl side chains (which impart a “kink” to the molecule), PE forms a conical shape.^2^ This molecular structure is key to sculpting the curvature of the inner layer of cellular and organellar membranes, where PE is quantitatively the most abundant type of PL.^3^

In organelles where a high degree of membrane curvature is critical for the translation of structure to function, such as in the cristae that form the inner mitochondrial membranes, increases in PE content can improve performance, while losses can result in dysfunction that is so critical as to be incompatible with cellular and organismal life.^4^ While this provides a direct connection between PE and the control of energy metabolism, recent studies demonstrate additional roles for this glycerolipid in the regulation of whole-body metabolic homeostasis that are both complex and overlapping. For example, PE is primarily synthesized in the Kennedy Pathway through the cytidine diphosphate (CDP)-ethanolamine pathway, where CTP:phosphoethanolamine cytidylyltransferase (Pcyt2) catalyzes the second and rate-limiting step. Because diacylglycerol (DAG) is a substrate in the third and final step of the PE-Kennedy pathway, limitations in Pcyt2 activity are associated with reduced utilization, and therefore increased accumulation of this bioactive lipid, with subsequent consequences for the risk of developing insulin resistance and obesity.^5^ At the same time, others have found that hepatic insulin signaling can be improved by reducing the ratio of PC:PE through a mechanism that is unrelated to DAG levels,^6^ but could involve other factors such as modulation of the efficiency of function of the sarco-endoplasmic reticulum calcium ATPase pump,^7^ which is responsible for more than 20% of energy expenditure overall, and up to half of resting energy use in skeletal muscle specifically.^8^

As the largest sink for insulin-stimulated glucose disposal, a central role for skeletal muscle in the regulation of whole body energy and glucose metabolism is well established. Questions remain, however, regarding the role of skeletal muscle PE levels and Pcyt2 activity in the health and metabolic function of this tissue. These questions are particularly perplexing, given that mice heterozygous for *Pcyt2* global ablation develop nonalcoholic steatohepatitis,^5^ while liver-specific deletion of this enzyme causes hepatosteatosis *without* liver injury or impaired hepatic insulin signaling,^9^ necessarily implicating extra-hepatic organ systems in the pathology.

## *Pcyt2^+/−^* Mice Have Dysregulated Skeletal Muscle Structure and Lipid Metabolism, and Exhibit Adipose Tissue Infiltration

In their recent paper, Grapentine and colleagues have investigated the skeletal muscle phenotype in mice with global *Pcyt2* heterozygous gene ablation (*Pcyt2^+/−^*), and their findings provide substantial new data linking PE synthesis in this tissue not only to muscle health but also to systemic glucose dysregulation.^3^ In the current work, the authors examined skeletal muscles from wildtype and *Pcyt2^+/−^* mice, which were used since homozygous loss of this gene is embryonic lethal. Most noticeably, the quality of skeletal muscle changed dramatically with Pcyt2 deficiency, highlighting the critical role of this enzyme in the structural integrity of this tissue. Changes included evidence of degeneration, including the appearance of hyper-eosinophilic cells and vacuolization, as well as disruptions visible under electron microscopy, which included disordering of myofilaments and sarcomeres, resulting in irregular or disrupted Z-lines. Not surprisingly, skeletal muscle tissues from *Pcyt2^+/−^* mice had higher levels of fibrosis and macrophage infiltration compared to tissues from wildtype littermates.

Elevated levels of triacylglycerol (TAG) were also found in skeletal muscle fibres from these mice, which was not unexpected, given the disruption to DAG utilization and concomitant rise in DAG content within the tissue. Increased lipogenesis, resulting from elevated Srebp1c activation and enhanced dephosphorylation of acetyl-coenzyme A (CoA) carboxylase, and reduced lipolysis resulting from reduced activation of hormone-sensitive lipase and reduced *Cpt1* levels, supported that both higher lipid synthesis and reduced breakdown were factors in the observed myosteatosis. What was surprising, however, was the observation of apparent infiltration of skeletal muscle with adipose tissue cells, rather than just intramyocellular lipid droplets, which appeared in clusters to give a “cloud-like” appearance characteristic of classical adipose tissue depots.

## *Pcyt2^+/−^* Mice Have Dysregulated Skeletal Muscle Glucose Metabolism

Lipotoxicity significantly impairs glucose handling in numerous models, so the authors studied glucose metabolism in skeletal muscle, and found a near doubling in of levels and activity of the DAG-activated negative insulin signaling regulator protein kinase c (Pkc)α,^10^ together with reduced contents of the insulin signaling mediators IRS1, p85-PI3k, and phosphorylated and total Akt, as well as an 87% reduction in the ratio of plasma membrane:total Glut4 protein content. Not surprisingly, this reduced soleus and gastrocnemius glucose uptake under both basal and stimulated conditions in *Pcyt2^+/−^* mice, in association with reduced glycogen synthesis, but enhanced glycogen content.

## What Does This Tell Us About the Role of Skeletal Muscle *Pcyt2^+/−^* in Liver Metabolic Dysregulation

Given that myosteatosis is closely associated with the pathogenesis of nonalcoholic fatty liver disease, to the extent that it is used as a prognostic indicator in patients with end-stage liver disease,^3^ the findings from Grapentine et al. strongly suggest that the age-dependent non-alcoholic steatohepatitis (NASH) and insulin resistance reported in global *Pcyt2^+/−^* mice^5^ (but not in liver-specific *Pcyt2* knockout mice^9^) originates, at least in part, from pathological changes within skeletal muscle. To explain the overall outcomes, the authors postulated a model of metabolic re-routing. They suggest that reduced activity of Pcyt2 in skeletal muscle results in DAG accumulation that activates Pkcα to impair insulin signaling and glucose uptake, while problems with mitochondrial oxidation and muscle function overall decrease fuel utilization. The authors suggest that this increased the diversion of glucose in skeletal muscle into nonoxidative pathways, such as lipogenesis, which caused inflammation that further inhibited insulin signaling, and together with elevated glycogen stores, also further limited the uptake and utilization by skeletal muscle of glucose. With this major glucose sink diminished, and the tissue inflamed and dysfunctional, the authors suggest that mice became glucose intolerant, and rerouted this excess glucose into the production of TAG in liver.

Results from this study highlight the importance of PE synthesis in skeletal muscle structure, function, and metabolic health, providing a significant and novel insight into the role of this enzyme and this bioactive lipid in this important tissue. One limitation, which the authors acknowledge, is the use of a global *Pcyt2^+/−^*mouse. Given the magnitude of effects observed in the current study, future work in mice deficient in *Pcyt2* specifically in skeletal muscle would provide an exciting and direct insight into the nature of skeletal muscle–liver crosstalk in hepatosteatosis development and related metabolic complications.
